# Expansion-Based Clearing of Golgi-Cox-Stained Tissue for Multi-Scale Imaging

**DOI:** 10.3390/ijms23073575

**Published:** 2022-03-25

**Authors:** Qing-Hong Shan, Xin-Ya Qin, Jiang-Ning Zhou

**Affiliations:** 1 Chinese Academy of Science Key Laboratory of Brain Function and Diseases, Division of Life Sciences and Medicine, School of Life Sciences, University of Science and Technology of China, Hefei 230026, China; shanqh@ustc.edu.cn (Q.-H.S.); qinxinya@ustc.edu.cn (X.-Y.Q.); 2 Center for Excellence in Brain Science and Intelligence Technology, Chinese Academy of Sciences, Shanghai 200031, China

**Keywords:** Golgi-Cox stain, tissue clearing, 3D visualization, immunostain, multi-round immunostain

## Abstract

Obtaining fine neuron morphology and connections data is extraordinarily useful in understanding the brain’s functionality. Golgi staining is a widely used method for revealing neuronal morphology. However, Golgi-Cox-stained tissue is difficult to image in three dimensions and lacks cell-type specificity, limiting its use in neuronal circuit studies. Here, we describe an expansion-based method for rapidly clearing Golgi-Cox-stained tissue. The results show that 1 mm thick Golgi-Cox-stained tissue can be cleared within 6 hours with a well preserved Golgi-Cox-stained signal. At the same time, we found for the first time that the cleared Golgi-Cox-stained samples were compatible with three-dimensional (3D) immunostaining and multi-round immunostaining. By combining the Golgi-Cox staining with tissue clearing and immunostaining, Golgi-Cox-stained tissue could be used for large-volume 3D imaging, identification of cell types of Golgi-Cox-stained cells, and reconstruction of the neural circuits at dendritic spines level. More importantly, these methods could also be applied to samples from human brains, providing a tool for analyzing the neuronal circuit of the human brain.

## 1. Introduction

Since Camillo Golgi developed the Golgi staining method in 1873 [1], Golgi staining has been the most commonly used method for neuron morphology analysis. After a century of development, several modifications of the Golgi staining method have been developed, such as the Golgi-Cox method, Golgi–Kopsch method, and rapid Golgi method [2]. Nevertheless, there are some limitations of Golgi stain. The stained tissue is dark and strongly reduces light transmission, limiting its application in thick sections. Sections that are 100–200 μm thick are commonly used for neuronal morphology analysis [3]. It is challenging to reconstruct thick Golgi-Cox-stained tissues in three dimensions. Another limitation of Golgi staining is that it stains neurons randomly and is usually not compatible with immunostaining [4], making it difficult to study the morphology of specific cell types.

In recent years, different tissue clearance methods were developed for 3D imaging of intact tissues, such as CLARITY, PACT, CUBIC, MAP, iDISCO, Scale, and SWITCH [5,6,7,8,9,10,11,12]. These tissue-clearing methods use different strategies to match the refractive index, thereby reducing the tissue’s scattering and absorption of light, and finally making the tissue appear transparent. By combining genetic labeling methods or immunostaining, tissue clearance technology can be used to analyze large-scale neuronal circuits [8,11,13]. However, genetic labeling methods tend to be costly, rely heavily on complex instrumentation and skills, and are not easy to use [14]. Immunostaining can conveniently label different neuronal markers. However, the morphology of the neurons labeled using immunostaining is usually incomplete.

Combining the characteristics of good neuronal morphology labeling via Golgi staining and the tissue clearing for 3D imaging is very meaningful for neuronal circuit reconstruction. Previous studies successfully used CLARITY and CUBIC to clear Golgi-Cox-stained tissues for 3D imaging [14,15], showing that 3D imaging of Golgi-Cox-stained tissue using tissue clearing is a feasible protocol. Other researchers also performed immunostaining on Golgi-Cox-stained tissue. After using the paraformaldehyde (PFA) fixed tissue for Golgi-Cox staining, researchers detected several antigens in Golgi-Cox-stained tissue with reasonable immunological specificity [16], indicating that the antigens can be preserved when using the PFA fixed tissue for a Golgi-Cox stain. However, immunostaining using cleared Golgi-Cox-stained tissue has not been reported. It is still unclear whether the cleared Golgi-Cox-stained tissue can be used for immunostaining.

We set our goal to develop an easy and effective method for rapidly clearing Golgi-Cox-stained tissues for three-dimensional (3D) imaging that is compatible with immunostaining. The expansion-based clearing method can expand tissue to several times of its original size by embedding the tissue with water-absorbing hydrogel, and is widely used for tissue clearing and high-resolution imaging [5,17,18]. We chose the magnified analysis of the proteome (MAP) method [5] for clearing and expanding the Golgi-Cox-stained tissue because this expansion-based tissue clearing method has a simple protocol and is compatible with multiple rounds of immunostaining. At the same time, we modified the reagent used in the MAP method to make it easier to perform 3D immunostaining. By combining reflective confocal imaging, two-photon imaging, and immunostaining, the cleared Golgi-Cox-stained tissue could be used for large-volume 3D imaging, immunostaining of specific cell types, multiple rounds of immunostaining, and the reconstruction of neural circuits. More importantly, these methods were also applied to samples from human brains, providing a tool to analyze the neuronal circuits of the human brain.

## 2. Results

### 2.1. Optimal Golgi-Cox Staining and Tissue Clearing Method for Rapid Clearing

The reagent for darkening influences the Golgi-Cox staining result [19]. Ammonium hydroxide (NH_4_OH) and sodium thiosulfate (Na_2_S_2_O_3_) treatments are widely used for darkening of the tissue sections impregnated after Golgi-Cox solution [15,20], while lithium hydroxide (LiOH) is used for darkening of the whole-mount tissues [19,21]. Different darkening solutions were tested. The whole mouse brains were impregnated in Golgi-Cox solution for 14 days and then divided into two hemispheres. One hemisphere was treated with NH_4_OH and Na_2_S_2_O_3_, the other hemisphere was treated with LiOH (Figure S1A). The surface of the LiOH-treated tissue was darker than the NH_4_OH-and-Na_2_S_2_O_3_-treated tissue (Figure S1B). After sectioning the tissue into slices, in the cortex layer, the brain treated with NH_4_OH and Na_2_S_2_O_3_ showed reduced stain intensity compared with the LiOH-treated tissue, and the dendritic spines were not stained (Figure S1C). Meanwhile, uniform and intense Golgi-Cox-stain-labeled neurons were obtained in the LiOH-treated tissue, and the dendrites and dendritic spine were clearly visualized (Figure S1D). The results showed that after Golgi-Cox solution impregnation, using lithium hydroxide to darken the whole-mount sample can obtain a stable and optimistic Golgi-Cox stain result.

After obtaining a stable large-volume Golgi-Cox staining method, we tried to clear the Golgi-Cox-stained samples. CLARITY or CUBIC methods were applied to clear the Golgi-Cox-stained tissues [15]. As shown in Figure S2A, the CLARITY treatment could obtain a good clearing effect for tissues without Golgi-Cox staining (Figure S2A). The Golgi-Cox-stained samples were black and had a strong absorbing effect on light. After the CLARITY treatment, it was still black and opaque (Figure S2B), indicating that CLARITY may not be suitable for clearing Golgi-Cox-stained tissue. MAP technology uses high-concentration acrylamide to cross-link tissues and can expand the tissues about fourfold after absorbing water, thereby expanding the molecular gaps inside biological tissues [5]. We found that the conventional MAP method (containing 30% (*w*/*v*) acrylamide) could be used for clearing and expansion of the tissue (Figure S2C). However, when 3D immunofluorescence staining was performed, positive staining was only observed on the surface layer (Figure S2D), probably due to the high hydrogel concentration; the antibodies struggled to penetrate the tissue. We then reduced the acrylamide concentration to 10% (*w*/*v*) and found that the solution retained the features of the clearing and expansion of the tissue (Figure S2C). By performing 3D immunostaining with modified MAP reagents treated tissue, uniform immunostaining was achieved, indicating that antibodies penetrated to the deep layer of the tissue (Figure S2E). Considering its advantages regarding rapid clearing and ease of performing three-dimensional immunostaining, we selected the concentration of 10% (*w*/*v*) acrylamide for the clearing and expansion of Golgi-Cox-stained tissues and named this reagent modified MAP (containing 10% (*w*/*v*) acrylamide, 0.05% (*w*/*v*) bis-acrylamide, 4% (*w*/*v*) PFA, 0.1% (*w*/*v*) VA044, and 5% (*w*/*v*) sodium acrylate, dissolved in PBS).

**Figure 1:**
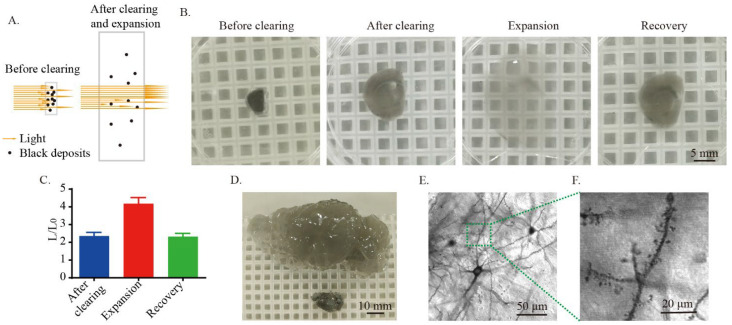
Rapid clearing of Golgi-Cox-stained tissue. (**A**) Schematic diagram showing that expansion of the Golgi-Cox-stained tissue increased light transmission. (**B**) Representative photos showing clearing, expansion, and recovery of 1 mm thick Golgi-Cox-stained tissue. The cleared samples were incubated in DI water for expansion. Otherwise, the samples were incubated in PBS. The 1 mm thick slices were cleared in the clearing solution for 6 h at 70 °C. (**C**) Statistics of the linear expansion of Golgi-stained tissue during clearing, expansion, and recovery. The histogram is shown as the mean ± SEM (n = 6). (**D**) Expansion of Golgi-Cox-stained hemibrain of mice and the cleared tissue was expended in DI water. The hemibrain was cleared in the clearing solution for 24 h at 70 °C. (**E**) The neuronal cell bodies and fibers after clearing and expansion. The image was taken using transmitted light with confocal microscopy. (**F**) The dendritic spines after clearing and expansion. The image was taken using transmitted light with confocal microscopy.

Next, we tried to test whether modified MAP could be used for the clearing of Golgi-Cox-stained tissue. We hypothesized that if the gap between the black deposits in the Golgi-Cox-stained tissue increases, the light absorption per unit area can be reduced and the tissue will become transparent (Figure 1A). By embedding the 1 mm thick Golgi-Cox-stained tissues with modified-MAP and allowing for 6 hours of clearing, the linear expansion of the tissue in PBS was about twofold (Figure 1B,C). When expanding with deionized (DI) water, the linear expansion was about fourfold, and the tissue was almost completely transparent (Figure 1B,C). At the same time, we also found that this clearing and expansion method could be used on mouse hemibrains (Figure 1D). To test whether the morphology of Golgi-Cox-stained neurons was preserved after clearing and expansion, we imaged the cleared tissue with confocal microscopy using transmitted light. Golgi-Cox-stained cell bodies and dendritic spines were well preserved (Figure 1E,F), indicating that modified MAP can be used for clearing Golgi-Cox-stained tissue.

### 2.2. Volume Imaging of Cleared Golgi-Cox-Stained Tissue with Confocal Reflection Mode

**Figure 2:**
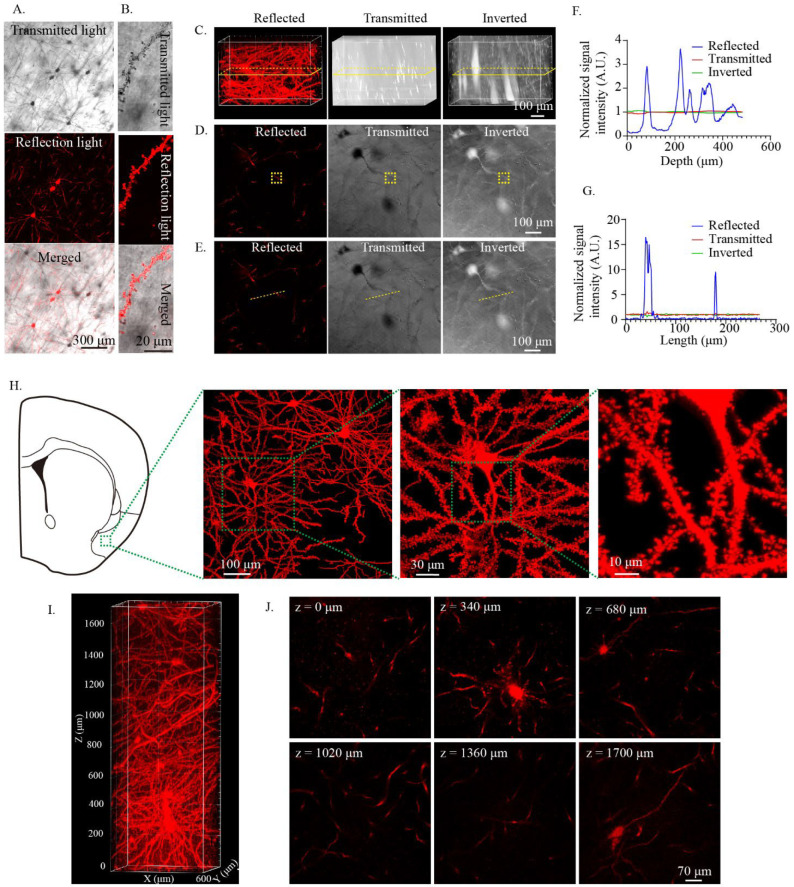
Three-dimensional imaging of cleared Golgi-stained tissue using the confocal reflective mode. (**A**) Imaging of the cleared Golgi-stained neuronal cell bodies and fibers using transmitted light and reflection light with confocal microscopy. (**B**) Imaging of the Golgi-stained dendritic spines after clearing using transmitted light and reflected light with confocal microscopy. (**C**–**G**) Imaging of cleared Golgi-Cox-stained tissue using confocal reflective mode increases spatial resolution. (**C**) Three-dimensional rendering of the same position of cleared Golgi-Cox-stained tissue with confocal reflective mode (reflected) and transmitted light (transmitted). The images obtained by transmitted light were inverted (inverted) and 3D rendered to facilitate visualization. The inserted yellow rectangle is the region of interest for the analysis of the signal intensity in (**D**,**E**). (**D**) Sectional image from (**C**). The inserted yellow rectangle was the region of interest for the analysis of the signal intensity through the z-axis. (**E**) Sectional image from (**C**). The inserted yellow line was the region of interest for the analysis of the signal intensity through xy plane. (**F**) Analysis of the normalized signal intensity through the z-axis. The normalized signal intensity was calculated with the formula: signal value/average signal value. (**G**) Analysis of the normalized signal intensity through the xy plane. The normalized signal intensity was calculated with the formula: signal value/average signal value. (**H**) Three-dimensional imaging of the fine morphology of cleared Golgi-stained tissue showing the neuronal cell bodies, fibers, and dendritic spines. The samples were cleared with modified MAP and incubated in PBS. The images were obtained with a 20 × 0.8 NA air objective lens. (**I**,**J**) Three-dimensional imaging of larger-volume cleared Golgi-stained tissue. (**I**) Three-dimensional rendering of the cleared Golgi-stained tissue. One millimeter thick slices were used for clearing and imaging (about 2 mm thick after clearing and incubating in PBS). (**J**) Sectional images at different depths.

Transmitted light is conventionally used for the imaging of Golgi-Cox-stained tissue. However, imaging with transmitted light results in low spatial resolution when performing three-dimensional imaging [22]. Since Golgi-Cox-stained tissue has the characteristic of reflecting light, the confocal reflection mode can be used to image Golgi-Cox-stained tissue to obtain high-resolution imaging [16,22,23]. First, we tested whether the Golgi-Cox-stained tissue after modified MAP clearing can be imaged using the confocal reflection mode. During testing, 300 μm thick cleared Golgi-Cox-stained tissue was used. The results showed that both transmitted light and reflected light can be used to image modified MAP-cleared Golgi-Cox-stained tissue. The cell body and dendrite were clearly seen under these two imaging conditions (Figure 2A). At high magnification, the dendritic spines of neurons were clearly seen (Figure 2B). Comparing transmitted light and reflected light for 3D imaging of the cleared tissue, we found that reflected light imaging had a better spatial resolution (Figure 2C–G). Subsequently, we used the confocal reflection mode to perform 3D imaging of cleared Golgi-Cox-stained tissue in the mouse piriform cortex. We found that the fine morphology of the neurons was well preserved, and the cell body, dendrites, and dendritic spines were clearly visible (Figure 2H). The results indicated that the confocal reflection mode can be used for high-resolution 3D imaging of the cleared Golgi-Cox-stained tissue. Subsequently, we tested the use of the confocal reflection mode to perform 3D imaging of large-volume cleared Golgi-Cox-stained tissue. During testing, 1 mm thick (about 2 mm thick after clearing and incubation in PBS) Golgi-Cox-stained tissues were used, and a 20× water immersion objective lens was used for imaging. We found that the imaging range of 1.7 mm depth could be clearly imaged (Figure 2I,J), indicating that the confocal reflective mode could also be used for the 3D imaging of large-volume cleared Golgi-Cox-stained tissues.

### 2.3. Volume Image of Cleared Golgi-Cox-Stained Tissue with Two-Photon Microscopy

**Figure 3:**
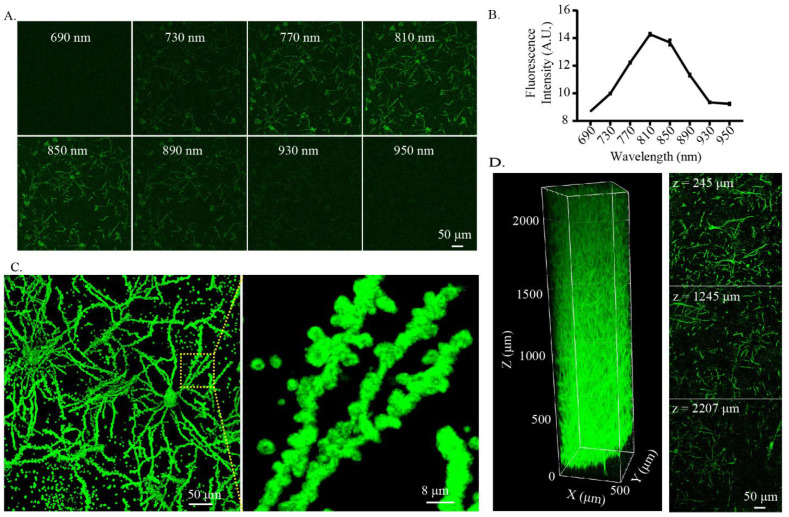
Three-dimensional imaging of cleared Golgi-stained tissue using two-photon microscopy. (**A**,**B**) Testing of the wavelength for excitation of the cleared Golgi-stained tissue. (**A**) The same position of the tissue was excited with 690–950 nm wavelengths with 40 nm interval and captured using an emission filter tuned to 495–540 nm. (**B**) Statistics of the fluorescence signal intensity of Golgi-stained samples under different excitation wavelengths. The plot is shown as the mean ± SEM (n = 3) (**C**) Three-dimensional imaging of the fine morphology of cleared Golgi-stained tissue using two-photon microscopy. The images were obtained with 810 nm excitation light and captured at 495–540 nm. The left panel shows the neuronal cell bodies and fibers after clearing and the right panel shows the dendritic spines. (**D**) Three-dimensional imaging of larger volume cleared Golgi-stained tissue using two-photon microscopy. Left panel: Three-dimensional rendering of the cleared Golgi-stained tissue. One millimeter thick slices were used for clearing and imaging (about 2 mm thick after clearing and incubating in PBS). Right panel: Sectional images at different depths.

Since mercury compounds within the Golgi-Cox-stained tissue are sensitive to photoexcitation and could be excited by a two-photon laser [15], we tested whether the cleared Golgi-Cox-stained tissue can be used for two-photon imaging. We used a two-photon laser (690–950 nm at 40 nm interval) for excitation, captured using an emission filter tuned to 495–540 nm to obtain the appropriate excitation light wavelength for two-photon imaging. The signal intensity of the Golgi-Cox-stained sample was the highest when excited using an 810 nm wavelength (Figure 3A,B). Thus, this wavelength was used for two-photon imaging. By imaging the cleared Golgi-Cox-stained tissue with two-photon microscopy, the cell bodies, neurites, and dendritic spines were clearly visible (Figure 3C). Subsequently, we tested the use of two-photon imaging to perform large-scale 3D imaging of the cleared Golgi-Cox-stained tissue. For the clearing and imaging, 1 mm thick (about 2 mm thick after clearing) Golgi-Cox-stained tissues were used. We found that the cleared Golgi-Cox-stained tissue could be imaged in the entire 2 mm imaging range (Figure 3D, Movie S1), indicating that two-photon imaging could be used to perform large-scale 3D imaging of the cleared Golgi-Cox-stained tissue.

### 2.4. Cleared Golgi-Cox Tissue Is Compatible with Immunostaining

**Figure 4:**
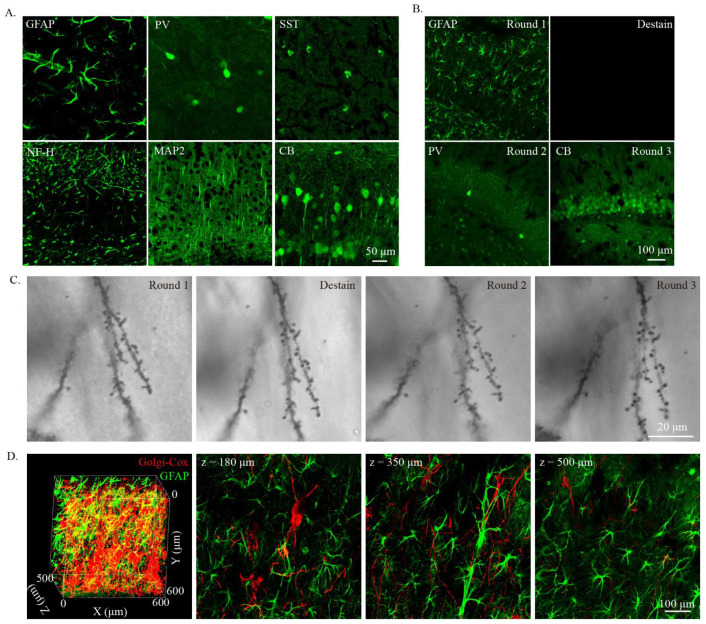
Immunostaining of cleared Golgi-stained tissue. (**A**) Immunostaining of Golgi-stained tissue with various antibodies. GFAP, glial fibrillary acidic protein; PV, parvalbumin; SST, somatostatin; NF-H, neurofilament 200; MAP2, microtubule-associated protein 2; CB, calbindin. (**B**) Multi-round immunostaining of the modified MAP cleared Golgi-Cox-stained tissue. Repeated staining and destaining of a single modified MAP cleared tissue. Mouse brain slices that were 100 μm thick were used. (**C**) The preservation of dendritic spines during multi-round immunostaining. The image was taken using transmitted light with confocal microscopy. (**D**) Three-dimensional immunostaining of cleared Golgi-stained tissue. Golgi-stained tissues that were 250 μm thick (about 500 μm thick after clearing) were immunostained with GFAP antibody. The Golgi-stained signal was observed with confocal reflective mode using a 594 nm laser and immunostain signal was observed with 488 nm excitation light. Three-dimensional rendering of the 3D immunostaining is shown in the left panel, and the sectional images are shown in the right three panels. The Golgi-Cox-stained signal was obtained using the confocal reflective mode of confocal microscopy, and the immunostained signal was obtained using the standard confocal imaging mode.

To extend the use of Golgi-Cox staining, we next investigated whether cleared Golgi-Cox-stained tissue was compatible with immunostaining. As PFA fixation is important for the immunostaining of Golgi-Cox-stained tissue [16,23], PFA-fixed brains were used for Golgi-Cox staining and then cleared with modified MAP. For the immunostaining test, 100 μm thick cleared Golgi-Cox-stained tissues were used. We selected a glial fibrillary acidic protein (GFAP) antibody to label the astrocyte; parvalbumin (PV), calbindin (CB), and somatostatin (SST) antibodies to label the different types of interneurons; and neurofilament 200 (NF-H) and microtubule-associated protein 2 (MAP2) to label cytoskeleton protein. All six of the tested antibodies worked well on the cleared Golgi-Cox-stained tissues (Figure 4A). Hydrogel embedding can fix proteins tightly and enable multi-round immunostaining [5,8]. We then performed multi-rounds immunostaining in the same slices and monitored the change in the dendritic spine labeled with the Golgi-Cox stain (Figure 4B,C). In the first round of immunostaining, we tested the GFAP antibody and found that it gave a good immunostaining result (Figure 4B). Then, we used harsh conditions (200 mM SDS, 200 mM NaCl, and 50 mM Tris in DI water; pH titrated to 9.0; 70 °C for 16 h) to destain the immunostained tissue. After destaining, the immunostaining signal fully disappeared (Figure 4B). In the second and third rounds of immunostaining, PV and CB antibodies were used for testing, and all gave good staining results (Figure 4B). Notably, the individual spine can be clearly monitored through the entire multi-round staining procedure (Figure 4C), indicating that cleared Golgi-Cox-stained tissue could be used for multi-round immunostaining without losing the Golgi-Cox-stained spine. Next, we tested whether the cleared Golgi-Cox-stained tissue could be used for 3D immunostaining in thick sections. Here, 250 μm thick (500 μm thick after clearing) Golgi-Cox-stained tissue was 3D immunostained with GFAP antibody for testing. The results show that the staining of GFAP was distributed in the entire imaging range (Figure 4D).

**Figure 5:**
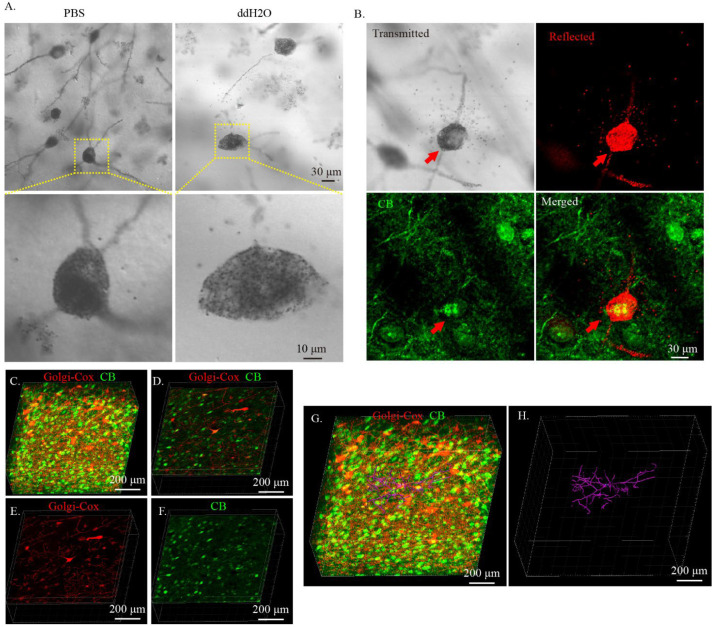
Reconstruction of specific neuron morphology in cleared Golgi-Cox-stained tissue by combining expansion and immunostaining. (**A**) Expansion of cleared Golgi-stained tissue with DI water. Cleared Golgi-stained tissues were incubated in either PBS or DI water. The image was obtained using transmitted light with confocal microscopy. (**B**) Imaging a single neuron labeled with Golgi-Cox stain and immunostaining (immunostained with calbindin antibody). The “transmitted” panel was obtained using transmitted light, The “reflected” panel was obtained using the confocal reflective mode, and the “CB” panel was obtained using the standard confocal imaging mode. (**C**–**H**) Three-dimensional reconstruction of a single neuron morphology labeled with both Golgi-stain and immunostain. The Golgi-Cox-stained signal was obtained using the confocal reflective mode of the confocal microscopy and the immunostained signal was obtained using standard confocal imaging mode. (**C**) Three-dimensional rendering showing the 3D immunostain of Golgi-Cox-stained tissue with CB. (**D**) Sectional image from (**C**) showing the colocation of Golgi-stained neuron with CB-immunopositive neuron. (**E**,**F**) The Golgi-stained channel and CB-stained channel of (**D**). (**G**) Reconstruction of the CB-immunopositive neuron morphology using the Golgi-Cox stain. (**H**) Reconstructed CB-immunopositive neuron. The reconstruction was performed using the FilamentTracer function of Imaris software and the neural fibers were tracked automatically by the software.

In our study, we found that after the expansion of the cleared Golgi-Cox-stained tissue in DI water, the black deposits on the surface of the Golgi-Cox-stained neurons were dispersed and permitted more light to transmit through Golgi-Cox-stained neurons (Figure 5A). We hypothesized that this feature would enable imaging of the immunostained signal and Golgi-Cox-stained signal in a single neuron. To verify this hypothesis, 300 μm thick cleared Golgi-Cox-stained slices were immunostained with anti-calbindin antibody and then expanded in DI water. Some CB immuno-positive neurons colocalized with Golgi-Cox-stained neurons were observed (Figure 5B). To reconstruct the morphology of CB immune-positive Golgi-Cox-stained neurons, 3D series imaging was obtained using confocal microscopy (Figure 5C–F). Taking advantage of the FilamentTracer function of Imaris software, the morphology of CB immune-positive neurons could be reconstructed by tracing the filament stained using the Golgi-Cox method (Figure 5G,H).

### 2.5. Reconstruction of the Neural Circuit at the Dendritic Spine Level

**Figure 6:**
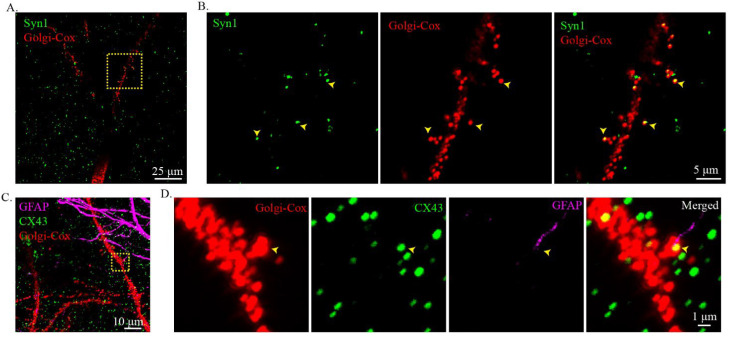
Reconstruction of the neuronal circuit at the dendritic spine level with the combination of Golgi-Cox staining and immunostaining. (**A**) Visualization of the connection between immunostain labeled pre-synaptic protein, Syn1, and Golgi-Cox-stain-labeled post-synaptic dendritic spines. The Golgi-Cox-stained signal was obtained using the confocal reflective mode of the confocal microscopy and the immunostained signal was obtained using standard confocal imaging. (**B**) Zoomed-in images from the insert in (**A**) showing the contact of the pre- and post-synaptic structures. The yellow arrows show the contact site. (**C**,**D**) Visualization of the connection between astrocytes and neurons. (**C**) Visualization of the immunostain labeled astrocyte (GFAP), gap junction (CX43), and Golgi-Cox-stain-labeled neural fibers. The Golgi-Cox-stained signal was obtained using the confocal reflective mode of the confocal microscopy and the immunostained signal was obtained using the standard confocal imaging mode. (**D**) Zoomed-in images from the insert in (**C**) showing the colocalization of the process of the astrocyte, CX43, and dendritic spine.

One fantastic feature of Golgi-Cox stain is that it can clearly stain the dendritic spine. To identify whether cleared Golgi-Cox-stained tissue can be used for high-resolution imaging, an antibody targeting synapsin 1 (Syn1) was used to label the pre-synaptic structure. After immunostaining, Syn1 was clearly seen in the cleared Golgi-Cox-stained slices (Figure 6A). At high magnification, colocalization of Syn1 and Golgi-Cox-stained dendritic spines was observed (Figure 6B). It is well established that astrocytes express connexin 43 (CX43), which is concentrated at gap junctions between astrocytic processes surrounding neuronal somata and dendrites [24]. We immunostained cleared Golgi-Cox-stained tissue with GFAP and CX43. The results showed that both GFAP and connexin 43 staining worked well on cleared Golgi-Cox-stained tissue (Figure 6C). At high magnification, the astrocyte process, connexin 43, and Golgi-Cox-stained dendritic spines were found to be close to each other (Figure 6D, Movie S2), indicating a gap junction between the dendritic spine and the process of astrocytes.

### 2.6. Imaging of Single-Cell Morphology of the Human Brain Using Cleared Golgi-Cox-Stained Tissue

**Figure 7:**
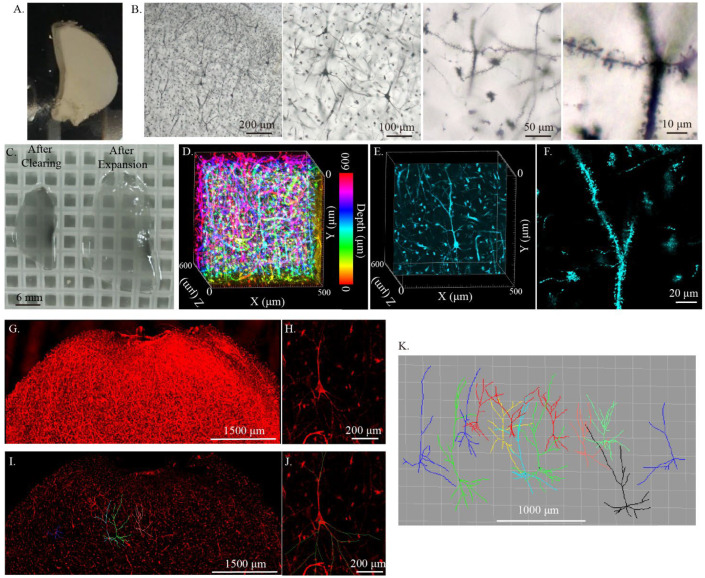
Three-dimensional imaging and reconstruction of modified MAP cleared Golgi-Cox-stained human brain tissue. (**A**) Whole-mount Golgi-Cox staining of human brain tissue. (**B**) Photomicrographs illustrating the fine neural structure, such as neural cell bodies, fibers, and dendritic spine labeled using Golgi-Cox staining. (**C**) Golgi-Cox-stained human brain tissue that was 300 μm thick (about 600 μm thick after clearing) after clearing and expansion. (**D**) Three-dimensional imaging of cleared Golgi-Cox-stained human brain tissue with confocal reflective mode and depth color coding. (**E**) Sectional image from (**D**) showing the fine neuronal morphology. (**F**) The dendritic spine in human brain tissue. The tissues were labeled using Golgi-Cox stain and imaged with confocal reflective mode. (**G**) Maximum intensity projection image showing larger volume imaging of the cleared Golgi-Cox-stained human brain tissue. (**H**) Sectional image from (**G**) showing an individual neuron. (**I**) Sectional image from (**G**) showing the reconstruction of neuronal morphology in human brain tissue. (**J**) Illustration of the reconstruction of the neuron morphology in (**H**). (**K**) The morphology of 13 neurons in the cortex of human brain tissue. The neuron morphology was reconstructed from the dataset of (**G**).

Our results showed that cleared Golgi-Cox-stained tissue could be used to reconstruct the neuronal morphology and circuit in thick mouse brain tissue; therefore, we tried to test this method in human brain tissues. After the Golgi-Cox solution impregnation, we found that using lithium hydroxide to darken the tissue from the human brain could obtain a good Golgi-Cox stain result (Figure 7A,B). When the Golgi-Cox-stained tissue was cleared using modified MAP and expanded, the tissue became transparent (Figure 7C). Subsequently, we used the confocal reflection mode to 3D imaging the cleared Golgi-Cox-stained human brain tissue. The results showed that neurons’ cell bodies and dendritic spines were clearly visible after clearing (Figure 7D–F). Subsequently, 6 mm × 3.2 mm × 0.5 mm of cleared Golgi-Cox-stained human brain tissue was imaged with the confocal reflection mode and Imaris software was used to reconstruct the neuron morphology (Figure 7G–K). The results showed 13 neurons manually reconstructed from the human brain cortex, indicating that cleared Golgi-Cox-stained human brain tissue could be used for reconstructing the morphology of large-volume human brain tissue.

**Figure 8:**
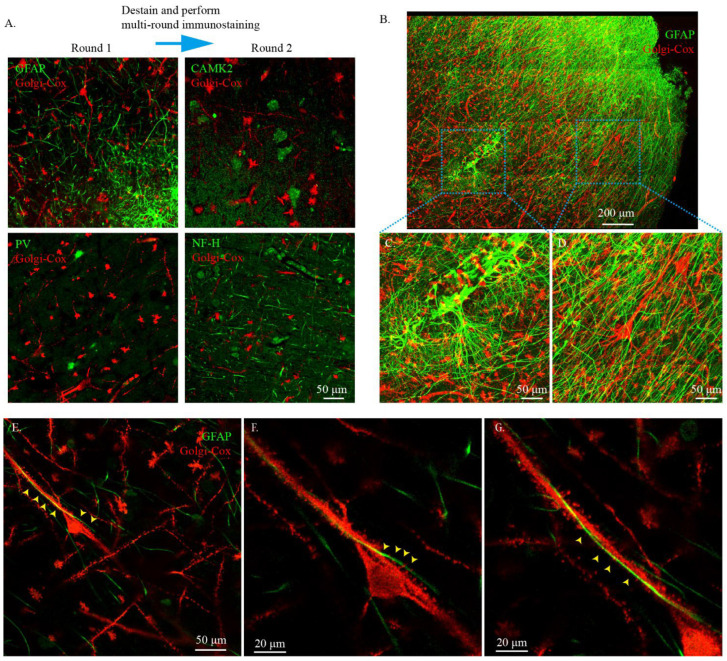
Immunostaining of cleared Golgi-Cox-stained human brain tissue. (**A**) Multi-round immunostaining of cleared Golgi-Cox-stained human brain tissue. The tissues were immunostained with GFAP and PV. After imaging, destaining was performed and the tissues were used repeatedly. CAMK2 and NF-H were stained in the next immunostain round. The Golgi-Cox-stained signal was obtained using the confocal reflective mode of the confocal microscopy, and the immunostained signal was obtained using the standard confocal imaging mode. (**B**) Three-dimensional immunostaining of cleared Golgi-Cox-stained tissue with GFAP. The Golgi-Cox-stained signal was obtained using the confocal reflective mode of the confocal microscopy, and the immunostained signal was obtained using the standard confocal imaging mode. (**C**) Zoomed-in image from the insert in (**B**) showing the fine morphology of astrocyte labeled using the immunostain. (**D**) Zoomed-in image from the insert in (**B**) showing the fine morphology of neurons labeled using the Golgi-Cox stain. (**E**) High-resolution imaging of neuron–neuron and neuron–astrocyte connections in the human brain. The yellow arrows indicate a neuronal cell body in close contact with other neuron fibers and the process of astrocytes closely surrounding the neuron fibers. The Golgi-Cox-stained signal was obtained using the confocal reflective mode of the confocal microscopy and the immunostained signal was obtained using the standard confocal imaging mode. (**F**,**G**) Zoomed-in images from (**E**) showing the process of astrocytes closely surrounding the fiber of the neuron.

To examine whether the cleared Golgi-Cox-stained human brain tissues could be used for immunostaining and multiple-round immunostaining, 100 μm thick cleared Golgi-Cox-stained human brain slices were used for testing. The slices were started with immunostaining with GFAP and PV. The results showed that the processes of astrocytes labeled using GFAP and the cell bodies of interneurons labeled using PV could be clearly seen (Figure 8A). Subsequently, we destained the tissue and then immunostained the same slices with excitatory neuron marker CAMK2 and the nerve fiber marker NF-H. The cell bodies of excitatory neurons labeled using CAMK2 and the fiber of neurons labeled using NF-H could also be clearly seen (Figure 8A). The results showed that the cleared Golgi-Cox-stained tissue was compatible with immunostaining and multi-round immunostaining. Next, we performed 3D immunostaining on the cleared Golgi-Cox-stained human brain samples. The GFAP antibody was used to label astrocytes to observe their 3D connections with neurons. The morphology of immunostained astrocytes and Golgi-Cox-stained neurons was clearly observed (Figure 8B–D). At high magnification, a neuronal cell body was in close contact with other neuron fibers, and a process of astrocytes closely surrounded the neuron fibers (Figure 8E–G, Movie S3), indicating neuron–neuron and neuron–astrocyte connections.

## 3. Discussion

We developed a novel pipeline for the expansion-based clearing and multi-round immunostaining of Golgi-Cox-stained tissue (the flow chart of the pipeline is shown in Figure S4 and the comparison between the current method and other Golgi-Cox stain and tissue clearing methods is provided in Table S1). The pipeline used LiOH to replace NH_4_OH and Na_2_S_2_O_3_ for darkening the Golgi-Cox-solution-impregnated tissue to obtain a stable and good Golgi-Cox staining result. The modified MAP method, which is more suitable for 3D immunostaining, was used for the clearing of Golgi-Cox-stained tissue. The advantages of the modified-MAP-based Golgi-Cox-stained tissue clearing method include a fast processing speed, well-preserved Golgi-Cox-stained signal, and compatibility with immunostaining and multi-round immunostaining. Most importantly, this pipeline can be applied to samples from human brains. Combining Golgi-Cox staining with tissue clearing, immunostaining, and multi-round immunostaining, Golgi-Cox-stained tissue could be used for the 3D analysis of neuron morphology, reconstruction of the morphology of specific neuronal types, and even mapping the neuronal circuits at the dendritic spine level.

Previous studies have used Golgi-Cox-stained tissue for tissue clearing or immunostaining [15,16]. The darkening solution they used was an ammonia solution and sodium thiosulfate. These two reagents are commonly used to develop thin Golgi-Cox-stained slices (usually 50–200 μm) and gave a good result with short-term treatment (10–20 min treatment). However, short-term ammonia solution and sodium thiosulfate treatment are not suitable for larger-volume Golgi-Cox-stained tissue, as the time is not sufficient for complete penetration of the reagents into the tissue. When the treatment duration is increased (2 h treatment), the Golgi-Cox-stained signal is bleached by long-term sodium thiosulfate treatment [25]. Lithium hydrate is first used in tungstate modification of the Golgi-Cox method [26] and then used to darken Golgi-Cox-solution-treated brain and gives better staining than the ammonia-solution-treated brain [19,21]. In our experiment, we found that lithium hydrate treatment in both fresh and PFA-fixed brains gave satisfactory Golgi-Cox stain results. We concluded that lithium hydrate was a suitable reagent for darkening whole mount Golgi-Cox-stained tissues.

Since the Golgi stain solution impregnates neurons in a random manner, the immunostaining is therefore used to label specific cells [16]. However, only the surface (about 20–30 μm) of the slices can be stained with antibodies, which is not suitable for 3D visualization of Golgi and immunostained signals. In addition, the Golgi-Cox-stained neurons were dark and prevent light from being transmitted through the neurons, making it difficult to perform the 3D imaging of the Golgi-Cox-stained tissue. Here, we showed that after hydrogel-embedding and clearing, the lipid of the tissue was removed, and the protein was preserved. Consequently, the Golgi-Cox-stained tissue was transformed into a light- and antibody-accessible form and was compatible with 3D immunostaining and imaging. The cleared Golgi-Cox-stained tissue could also be used for multi-round immunostaining. Combined with multi-round immunostaining, several different proteins could be detected in a single cleared Golgi-Cox-stained tissue, and the distribution of different cell markers and the morphology of Golgi-Cox-stained neurons in Golgi-Cox-stained tissues could be reconstructed.

As the work distance of a high-power objective lens is usually very short, obtaining a larger volume of high-power images is usually very difficult. For low-power objective lenses, the work distance can be extremely long (e.g., 8 mm work distance for 25× objective lens) [5]. This would be very useful for high-resolution 3D imaging of large-volume expanded tissue with a low-power objective lens. The cleared Golgi-Cox-stained tissue can expand about four-fold when incubated in DI water. The resolution of images acquired at 25× magnification of expanded tissue is theoretically identical to 100× magnification when acquiring unexpanded tissue, which is sufficient to observe the dendrite spine at a large volume. We hypothesize that by using a long work distance objective lens, the connections of the larger volume Golgi-Cox-stained tissue can be reconstructed at the dendritic spine level.

The most prominent feature of the Golgi stain is that it can stain the full dendrites of a single neuron. Mapping the neural circuit at the dendritic spine level is an attractive perspective for understanding the connections between complex nervous systems. We found that connections between neurons of both mouse brain and human brain could be observed in Golgi-stained samples (Figure S3), indicating that the cleared Golgi-Cox-stained tissue can be used for studying the neuronal circuits. At the same time, we also found that the cleared Golgi-Cox-stained tissue was compatible with immunostaining, 3D immunostaining, and multi-round immunostaining. The cell type of Golgi-Cox-stained neurons could be identified by using immunostaining. Therefore, we believe that by combining Golgi-Cox staining with multi-round immunostaining, the cell types of interconnected Golgi-Cox-stained neurons can be identified, meaning that specific types of neuronal circuits in the mouse or human brain can be obtained by using Golgi-Cox-stained tissue.

The method of expansion base rapid clearing of Golgi-Cox-stained tissue has the potential of expanding the application of Golgi stain from neural morphology analysis to neural circuit reconstruction. With its simplicity and broad applicability, we suggest that the newly developed pipeline for clearing Golgi-Cox-stained tissue may complement existing methods and enable new approaches in studying neural systems.

## 4. Materials and Methods

### 4.1. Experimental Model and Subject Details

A total of 30 adult male (n = 24) and female (n = 6) C57BL/6 mice weighing approximately 20–25 g were used in the development of these methods. The animals were split into the replication of the CLARITY (n = 6), MAP (n = 3), and modified MAP (n = 3) tissue, testing Golgi-Cox stain parameter tissue (n = 3), and 3D imaging and immunostaining tissue (n = 15). Animals were housed in groups of 2–4 in 12 h light/12 h dark cycles, with food and water provided ad libitum. All animal experiments and procedures were approved by the Animal Care and Use Committee of the University of Science and Technology of China.

### 4.2. Human Tissue Treatment

Human tissue was obtained from a surgical patient. The sample was putatively healthy tissue from surgical corridors of temporal lobe resections for epilepsy treatment. Before the Golgi-Cox stain, the tissue was fixed in 10% neutral formaldehyde for 2 weeks at room temperature. All the Golgi-Cox staining, clearing, and immunostaining procedures were performed in the same way as in the mouse brains.

### 4.3. Golgi-Cox Stain

The Golgi-Cox staining procedure was modified from that previously described [3]. The Golgi-Cox solution was prepared by mixing 50 mL of 5% (*w*/*v*) potassium dichromate (K_2_Cr_2_O_7_) solution, 50 mL of 5% (*w*/*v*) mercuric chloride (HgCl_2_), 40 mL of 5% (*w*/*v*) potassium chromate (K_2_CrO_4_), and 100 mL of DI water. After mixing the solutions, the bottle needed to be covered with aluminum foil to protect them from light and kept at room temperature for 48 h before use to allow for precipitate formation. The solution may be stored for up to 6 months.

The animals were deeply anesthetized with pentobarbital sodium and transcardially perfused with either PBS followed by 4% paraformaldehyde (PFA), or only with PBS. Brains were removed and then treated differently according to the purpose of the experiment. For brains perfused with PFA, the brain was immersed in 20 mL PFA for post-fixation for 24–48 h at 4 °C and then immersed in 20 mL Golgi-Cox solution in the dark at room temperature. For brains perfused with PBS, the brain was directly immersed in 20 mL Golgi-Cox solution in the dark at room temperature. After 24 h of immersion in Golgi-Cox solution, the solution of all the brains was refreshed with another 20 mL Golgi-Cox solution and left for 14 days at room temperature. Brains were then washed with DI water for 24 h, changing the DI water twice to wash away residual Golgi-Cox solution. After washing, the tissues were darkened with different reagents. For the lithium hydrate (LiOH)-treated group, the brain was darkened by means of immersion in 1% LiOH for 24 h to obtain a uniform stain result. For the ammonia-solution-treated group, the brain was darkened by means of immersion in 3:1 ammonia solution for 2 h, washing with DI water three times for 1 h each, then immersed in 10% sodium thiosulfate (Na_2_S_2_O_3_) for 2 h. After darkening, all the brains were washed in PBS for 24 h, and then we proceeded to the next step.

### 4.4. Hydrogel Embedding

Three hydrogel solutions were used for the hydrogel embedding of the tissue and the solutions were prepared freshly before incubation. The formulations of the three hydrogel solutions are described below:

CLARITY: This solution contained 4% (*w*/*v*) acrylamide, 0.05% (*w*/*v*) bisacrylamide, 4% (*w*/*v*) PFA, and 0.25% (*w*/*v*) VA-004, dissolved in PBS.

MAP: This solution contained 30% (*w*/*v*) acrylamide, 0.05% bis-acrylamide (*w*/*v*), 4% (*w*/*v*) PFA, 0.1% (*w*/*v*) VA044, and 5% (*w*/*v*) sodium acrylate, dissolved in PBS. 

Modified-MAP: This solution contained 10% (*w*/*v*) acrylamide, 0.05% (*w*/*v*) bis-acrylamide, 4% (*w*/*v*) PFA, 0.1% (*w*/*v*) VA044, and 5% (*w*/*v*) sodium acrylate, dissolved in PBS.

Similar operation steps were used for hydrogel embedding of the tissues with or without Golgi-Cox staining. The tissues were immersed in hydrogel solution for 48 h at 4 °C to ensure uniform chemical diffusion into the sample. Following the diffusion step, hydrogel embedding was performed by incubating the samples in a water bath at 37 °C for 3 h to polymerize the CLARITY hydrogel solution, or at 50 °C for 3 h to polymerize the MAP or modified MAP hydrogel solution. The hydrogel-embedded tissues were then cut into 50 to 1000 µm thick slices using a vibratome (VT1200S, Leica).

### 4.5. Tissue Clearing

For CLARITY-treated tissues, the tissues were then cleared by incubating in sodium borate buffer (200 mM, pH 8.5) containing 4% (*w*/*v*) sodium dodecyl sulfate (SDS) at 37 °C. Generally speaking, the CLARITY-treated whole brain needed 4 weeks and the 1 mm thick slices needed 1 week for clearing. For MAP- and modified-MAP-treated tissue, the slices were collected and incubated in the clearing solution (200 mM SDS, 200 mM NaCl, and 50 mM Tris, pH 9.0) at 70 °C for 3–12 h to clear the tissue. For the 50 to 300 µm thick slices, 3 h incubation was enough to obtain an excellent clear result. For slices thicker than 300 µm, long-term incubation could be applied (usually 6 h for 1 mm thick slices and 24 h for hemibrains). Cleared Golgi-Cox-stained tissues were washed in PBS with 1% (*v*/*v*) Triton X-100 (PBST) five times for 1 h each to wash away residual SDS, as SDS would inhibit the immunostaining process. After washing, the cleared tissues were incubated in PBS and stored at 4 °C for further use.

### 4.6. Immunostaining of Cleared Tissue

Cleared Golgi-Cox-stained slices were incubated with blocking buffer (5% normal donkey serum in PBST) at 37 °C for 12 h, then incubated with primary antibodies (typical dilution, 1:100–1:200) in blocking buffer at 37 °C for 24 h, followed by washing at 37 °C for 2 h in PBST for three times. The tissues were then incubated with secondary antibody (typical dilution, 1:200) in blocking buffer at 37 °C for 24 h, followed by washing at 37 °C for 2 h in PBST three times. For the multi-round immunostaining, samples were incubated in the clearing solution for 12–16 h at 70 °C to destain. After destaining, the samples were washed with PBST at 37 °C for 1–2 h three times before being processed in the next round of immunostaining. The antibodies used are listed in Table S2.

### 4.7. Imaging and Analysis

The mounting procedure was similar to the previous descriptions [5]. Briefly, samples were placed on a Petri dish or a glass-bottomed Willco dish filled with PBS or DI water, and the samples were stabilized for at least 1 h before imaging. The samples were imaged with the Olympus FV1200-MPE multiphoton laser scanning confocal microscope system or Zeiss LSM 710 confocal microscope. For one-photon imaging, 488, 594, and 405 nm lasers were used to image the fluorescence-labeled immunostaining signal, and a 594 nm laser was used for reflective confocal imaging. For multiphoton imaging, a 690–950 nm laser was used for excitation. The images were visualized and analyzed with Fiji or Imaris (Bitplane Imaris, Oxford, UK).

## Data Availability

The data that support the findings of this study are available from the corresponding author upon reasonable request.

## Supplementary Materials

The following supporting information can be downloaded at: https://www.mdpi.com/article/10.3390/ijms23073575/s1.
