# Supplementary material for: Expansion-Based Clearing of Golgi-Cox-Stained Tissue for Multi-Scale Imaging
Source: Int J Mol Sci. 2022 Mar 25;23(7):3575. doi: 10.3390/ijms23073575 (PMC8998187; doi:10.3390/ijms23073575)
Supplement: Supplementary file 1 [file ijms-23-03575-s001.zip › ijms-1617287-supplementary.pdf]

Expansion Based Clearing of Golgi-Cox Stain Tissue for Multi-Scale Imaging

Supplementary Information

Authors: Qing-Hong Shan, Xin-Ya Qin, Nan Zhou, Xue-Yan Wu, Jiang-Ning Zhou

Supplementary Figures

Supplementary Table

Supplementary Videos

Supplementary Figure S1: Whole-mount darkening of Golgi-Cox solution impregnated mouse brain.

Supplementary Figure S2: Tissue clearing with modified-MAP method.

Supplementary Figure S3: Interconnections between Golgi-Cox stained nerve fibers.

Supplementary Figure S4: Flow chart showing the main steps for clearing and immunostaining the Golgi-Cox stained tissue.

Supplementary Table S1: Compare current method with and Golgi-Cox stain and tissue clearing method.

Supplementary Table S2: List of antibodies used in this experiment.

Supplementary Video S1: 3D imaging of 2-mm-thick cleared Golgi-Cox stained tissue with two-photon microscopy

Supplementary Video S2: High-resolution 3D imaging of the connection between Golgi-Cox stained neuronal fibers and immunostain labeled astrocyte fibers. The cleared Golgi-Cox stained tissue is immunostained with GFAP and CX43. Astrocytes (labeled with GFAP) are shown in magenta, gap junction (labeled with CX43) are shown in green, and neuronal fibers (labeled with Golgi-Cox) are shown in red.

Supplementary Video S3: Three-dimensional observation of neuron-to-neuron and neuron-to-astrocyte connections in Golgi-Cox stained human brain tissue. The cleared Golgi-Cox stained tissue is immunostained with GFAP. Astrocytes (labeled with GFAP) are shown in green, and Golgi-Cox stained neurons are shown in red.

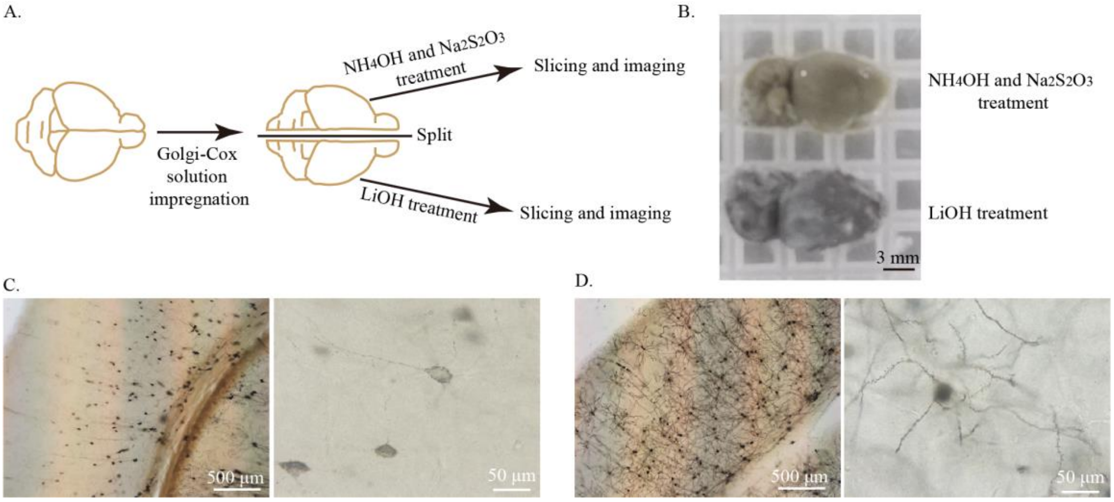

Figure S1. Whole-mount darkening of Golgi-Cox solution impregnated mouse brain. (A) Schematic diagram showing the procedure of darkening the two hemispheres from the same brain with the different darkening reagents. (B) The appearance of the two hemispheres darkens with the different darkening reagents. (C) Photomicrographs illustrating the result of whole-mount darkening hemispheres with long-term NH_4OH and $Na_2S_2O_3$ treatment. Left panel: low magnification images showing the cortex after darkening. Right panel: high magnification images showing the neurons and neuronal fibers after darkening. (D) Photomicrographs illustrating the result of whole-mount darkening hemispheres with long-term LiOH treatment. Left panel: low magnification images showing the cortex after darkening. Right panel: high magnification images showing the neurons and neuronal fibers after darkening.

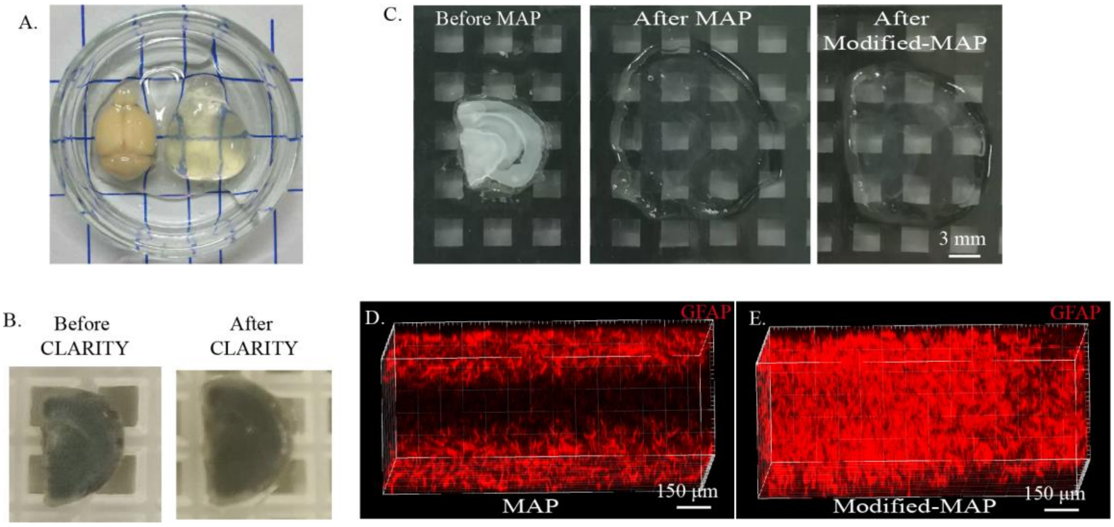

Figure S2. Tissue clearing with modified-MAP method. (A) Testing of the CLARITY method. (B) 1-mm-thick Golgi-Cox stained tissue before and after clearing with CLARITY. (C) 300-µm-thick slices treated with MAP and modified-MAP. (D) 3D immunostaining of MAP and modified-MAP treated tissue with GFAP antibody. The 3D renderings showed vast increase in uniformity of labeling after modified-MAP treatment.

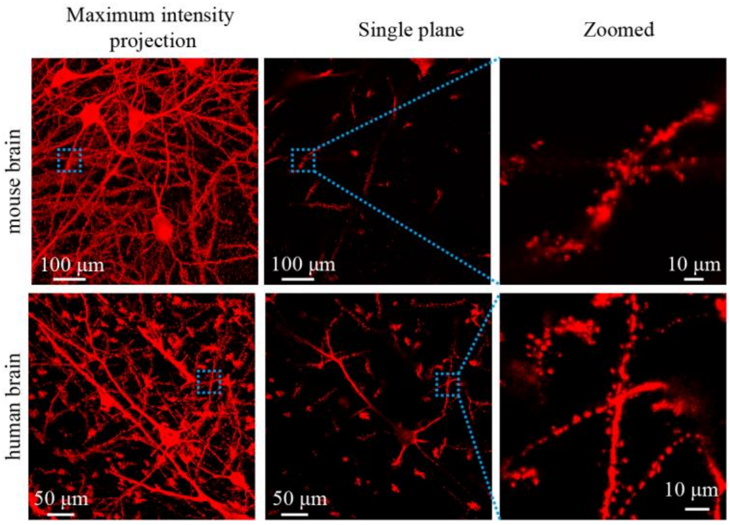

Figure S3. Observation of interconnections between nerve fibers in Golgi-Cox stained mouse and human brain tissue.

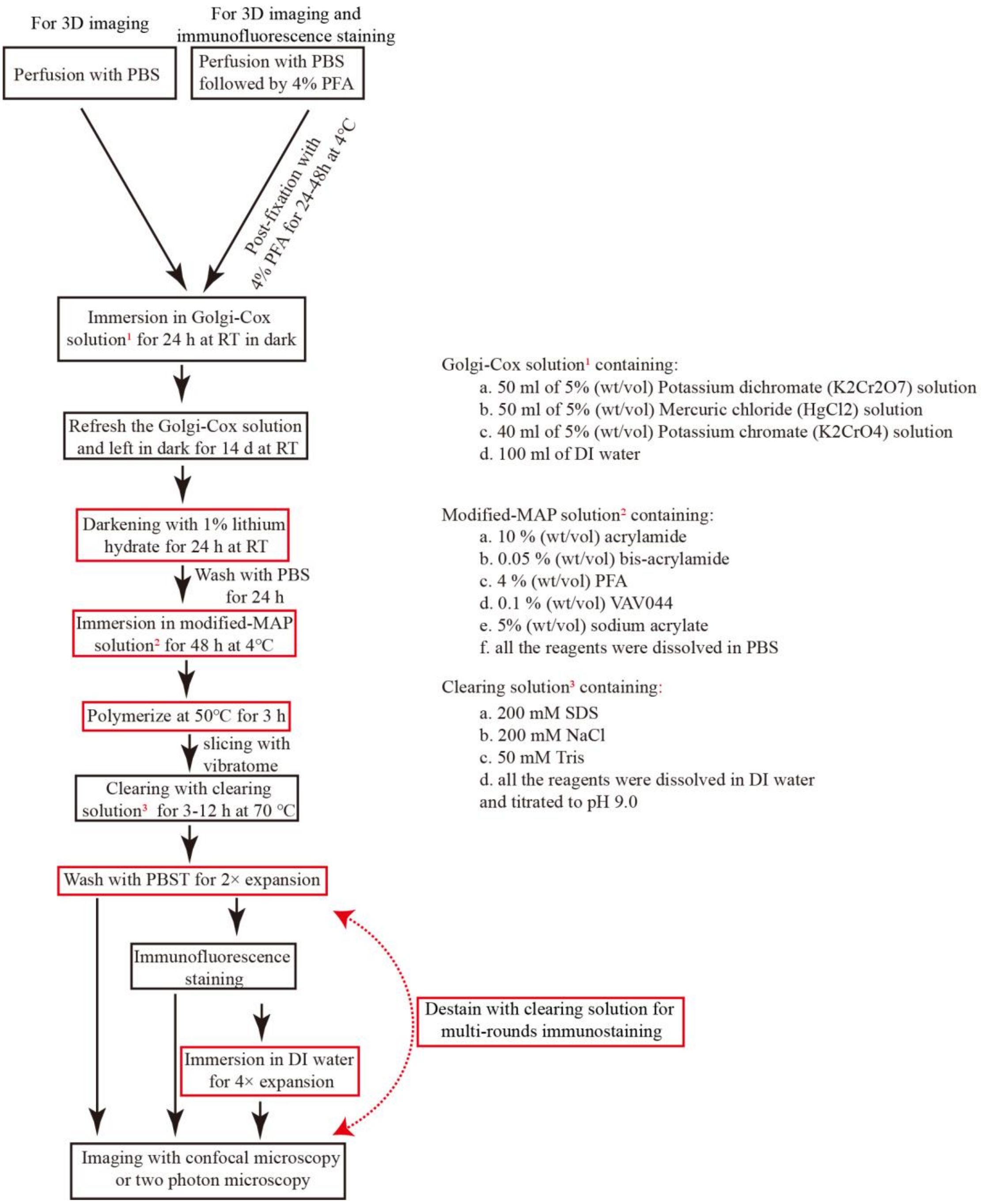

Figure S4: Flow chart showing the main steps for clearing and immunostaining the Golgi-Cox stained tissue. The steps marked in red boxes are the key steps for clearing, multi-rounds immunostaining, and 3D imaging of Golgi-Cox stained tissues. The solution recipes marked with red numbers in the flowchart are shown in the list on the right. These reagents are needed to be prepared for clearing and performing multi-rounds immunostaining of Golgi-Cox stained tissue. Note: RT, room temperature.

Table S1: Compare current method with other Golgi-Cox stain and tissue clearing method.

Methods	Golgi-Cox stain combined with modified MAP (current method)	Standard Golgi-CoX [1]	Golgi-Cox stain combined with CLARITY [2]	Golgi-Cox stain combined with CUBIC [3]	Standard MAP [4]
Tissue used	Fresh or PFA fixed	Fresh	PFA fixed	PFA fixed	PFA fixed
Impregnation solution	Standard Golgi-CoX solution	Standard Golgi-CoX solution	URG stock solution	Standard Golgi-CoX solution	N.T.
Darkening solution	Lithium hydroxide	Ammonium Hydroxide and Sodium Thiosulfate	Ammonium Hydroxide and Sodium Thiosulfate	Ammonium Hydroxide and Kodak Carestream Fixer solution	N.T.
Hydrogel solution	Modified MAP solution with low acrylamide concentration	N.T	CLARITY solution	N.T	Standard MAP solution
Hydrogel embedding	YES	NO	YES	NO	YES
Clearing solution	SDS solution	N.T	SDS solution	CUBIC solution	SDS solution
Clearing time	3-24 h	N.T.	48 h or less	10 min-8 days	6-72 h
Tissue expansion	YES, maximum 4× expansion	N.T	N.T	N.T	YES, maximum 4× expansion
Immunostain compatible	YES	N.T.	YES	N.T.	YES
3D immunostain	YES	N.T.	N.T.	N.T.	YES
Multi-round immunostain	YES	N.T.	N.T.	N.T.	YES

Note: N.T. , not tested

Table S2. List of antibodies used in this experiment.

Target	Vendor	Catalog	Host species	Clonality	Dilution	Fluorescence conjuction
Somatostatin	Santa Cruz	SC-55565	Mouse	monoclonal	1:100	
Parvalbumin	Abcam	ab11427	Rabbit	polyclonal	1:200	
Calbindin	Abcam	ab11426	Rabbit	polyclonal	1:200	
GFAP	DAKO	Z0334	Rabbit	polyclonal	1:200	
Synapsin1	Sigma	SAB4502904	Rabbit	polyclonal	1:200	
Connexin 43	Abcam	ab219493	Goat	polyclonal	1:200	
MAP2	Sigma	M2320	Mouse	monoclonal	1:100	
NF-H	Sigma	N5389	Mouse	monoclonal	1:200	
CAMK2	Santa Cruz	SC-13082	Rabbit	polyclonal	1:50	
Rabbit IgG	Jackson Immunoresearch	715-545-151	Donkey	polyclonal	1:200	Alexa fluor 488
Rabbit IgG	Jackson Immunoresearch	715-475-151	Donkey	polyclonal	1:200	Dylight 405
Goat IgG	Jackson Immunoresearch	705-545-147	Donkey	polyclonal	1:200	Alexa fluor 488
Mouse IgG	Jackson Immunoresearch	715-545-151	Donkey	polyclonal	1:200	Alexa fluor 488

Reference

1. Zaqout, S.; Kaindl, A. M., Golgi-Cox Staining Step by Step. Frontiers in neuroanatomy 2016, 10, 38.
2. Kassem, M. S.; Fok, S. Y. Y.; Smith, K. L.; Kuligowski, M.; Balleine, B. W., A novel, modernized Golgi-Cox stain optimized for CLARITY cleared tissue. Journal of neuroscience methods 2018, 294, 102-110.
3. Vints, K.; Vandael, D.; Baatsen, P.; Pavie, B.; Vernaillen, F.; Corthout, N.; Rybakin, V.; Munck, S.; Gounko, N. V., Modernization of Golgi staining techniques for high-resolution, 3-dimensional imaging of individual neurons. Scientific reports 2019, 9, (1), 130.
4. Ku, T.; Swaney, J.; Park, J. Y.; Albanese, A.; Murray, E.; Cho, J. H.; Park, Y. G.; Mangena, V.; Chen, J.; Chung, K., Multiplexed and scalable super-resolution imaging of three-dimensional protein localization in size-adjustable tissues. Nat Biotechnol 2016, 34, (9), 973-81.
